# Salt-inducible kinase 2 and -3 are downregulated in adipose tissue from obese or insulin-resistant individuals: implications for insulin signalling and glucose uptake in human adipocytes

**DOI:** 10.1007/s00125-016-4141-y

**Published:** 2016-11-02

**Authors:** Johanna Säll, Annie M. L. Pettersson, Christel Björk, Emma Henriksson, Sebastian Wasserstrom, Wilhelm Linder, Yuedan Zhou, Ola Hansson, Daniel P. Andersson, Mikael Ekelund, Eva Degerman, Karin G. Stenkula, Jurga Laurencikiene, Olga Göransson

**Affiliations:** 1 Protein Phosphorylation Research Group, Department of Experimental Medical Science, Lund University, BMC C11, Klinikgatan 28, 22242 Lund, Sweden; 2 Lipid Laboratory, Department of Medicine Huddinge, Karolinska Institutet, Stockholm, Sweden; 3 Glucose Transport and Protein Trafficking, Department of Experimental Medical Science, Lund University, Lund, Sweden; 4 Diabetes and Endocrinology, Department of Clinical Sciences Malmö, Lund University, Malmö, Sweden; 5 Surgery, Department of Clinical Sciences Lund, Lund University, Lund, Sweden; 6 Insulin Signal Transduction, Department of Experimental Medical Science, Lund University, Lund, Sweden

**Keywords:** Gene and protein expression, Glucose uptake, HOMA-IR, Inflammation, Obesity, Salt-inducible kinase, SIK2, SIK3, Weight loss

## Abstract

**Aims/hypothesis:**

Salt-inducible kinases (SIKs) are related to the metabolic regulator AMP-activated protein kinase (AMPK). SIK2 is abundant in adipose tissue. The aims of this study were to investigate the expression of SIKs in relation to human obesity and insulin resistance, and to evaluate whether changes in the expression of SIKs might play a causal role in the development of disturbed glucose uptake in human adipocytes.

**Methods:**

SIK mRNA and protein was determined in human adipose tissue or adipocytes, and correlated to clinical variables. SIK2 and SIK3 expression and phosphorylation were analysed in adipocytes treated with TNF-α. Glucose uptake, GLUT protein levels and localisation, phosphorylation of protein kinase B (PKB/Akt) and the SIK substrate histone deacetylase 4 (HDAC4) were analysed after the SIKs had been silenced using small interfering RNA (siRNA) or inhibited using a pan-SIK-inhibitor (HG-9-91-01).

**Results:**

We demonstrate that *SIK2* and *SIK3* mRNA are downregulated in adipose tissue from obese individuals and that the expression is regulated by weight change. *SIK2* is also negatively associated with in vivo insulin resistance (HOMA-IR), independently of BMI and age. Moreover, SIK2 protein levels and specific kinase activity display a negative correlation to BMI in human adipocytes. Furthermore, SIK2 and SIK3 are downregulated by TNF-α in adipocytes. Silencing or inhibiting SIK1–3 in adipocytes results in reduced phosphorylation of HDAC4 and PKB/Akt, less GLUT4 at the plasma membrane, and lower basal and insulin-stimulated glucose uptake in adipocytes.

**Conclusion/interpretation:**

This is the first study to describe the expression and function of SIKs in human adipocytes. Our data suggest that SIKs might be protective in the development of obesity-induced insulin resistance, with implications for future treatment strategies.

**Electronic supplementary material:**

The online version of this article (doi:10.1007/s00125-016-4141-y) contains peer-reviewed but unedited supplementary material, which is available to authorised users.

## Introduction

The salt-inducible kinases (SIKs)—SIK1, SIK2 and SIK3—are related to AMP-activated protein kinase (AMPK), a master regulator of cellular and whole body energy homeostasis [1]. AMPK and AMPK-related kinases share sequence homology in their kinase domains [2] and are all activated by liver kinase B1 (LKB1) [3]. SIK1 was first identified in the adrenal glands of rats fed a high salt diet [4] and has since been described in several cell types [5–8]. SIK2 is highly expressed in adipose tissue and increases during adipocyte differentiation [9–11], while SIK3 displays a more ubiquitous expression [12].

Together SIKs control diverse cellular processes including the regulation of glucose and lipid metabolism in rodent liver [13–18] and adipose tissue [10, 19, 20]. Recently, it was shown that mice with global SIK2 deficiency display multiple defects in adipocyte metabolism [20]. Moreover, a previous study described increased expression and activity of SIK2 in white adipose tissue (WAT) from obese *db/db* mice [9], suggesting a role for SIK2 in obesity and diabetes. SIK3 has also been linked to metabolism, and *Sik3* ^-/-^ mice display disturbed lipid and glucose homeostasis [14, 17]. Furthermore, genetic variations in *SIK3* have been associated with dyslipidaemia and obesity in humans [21].

SIKs regulate gene expression by controlling the phosphorylation of transcriptional regulators, such as class II histone deacetylases (HDACs) [6, 22] and cAMP-response element binding protein (CREB)-regulated transcription co-activators (CRTCs) [23, 24]. This has, so far, mainly been studied in the liver but we recently demonstrated that HDAC4, CRTC2 and CRTC3 are direct substrates of SIK2 in rodent adipocytes [19]. In addition to the activating phosphorylation by LKB1, SIK2 is phosphorylated at several residues—Ser343, Ser358, Thr484 and Ser587—in response to cAMP/protein kinase A (PKA)-signalling in adipocytes, resulting in a subcellular relocalisation of SIK2 without altering kinase activity [19, 25]. Similarly, SIK3 is phosphorylated at several residues in response to cAMP/PKA in adipocytes [26].

The biological role of SIK2 in adipocytes is not fully understood and has almost exclusively been studied in rodents. Based on these studies, SIK2 appears to be required for GLUT4 expression and glucose uptake in adipocytes [19, 20]. Therefore, the aim of our study was to investigate the expression of SIKs, in particular SIK2, in adipose tissue from healthy and obese or insulin-resistant humans, and to analyse the importance of SIKs for glucose uptake in human adipocytes.

## Methods

### Clinical cohorts

Three different cohorts were used. Cohort 1 was used for *SIK2* and *SIK3* mRNA expression analysis in subcutaneous WAT and consisted of 56 individuals; of which, the 37 obese participants underwent gastric bypass surgery and 19 were non-obese controls [27]. Briefly, participants came to the laboratory after overnight fasting. Height, weight and waist circumference were measured and BMI calculated. Venous blood samples were collected for analysis of glucose, insulin, triacylglycerol and cholesterols [28]. An abdominal subcutaneous adipose tissue biopsy was taken as previously described [29]. In vivo insulin resistance was assessed by hyperinsulinaemic–euglycaemic clamp [30] and HOMA-IR calculated with the following formula: (glucose [mmol/l] × insulin [pmol/l])/405. Participants who underwent gastric bypass surgery were examined preoperatively and two years post surgery. Cohort 2 was used for protein and kinase activity analysis, and absolute quantification of *SIK* mRNA in primary adipocytes. This cohort consisted of 23 individuals who underwent laparoscopic cholecystectomy or gastric bypass surgery. Subcutaneous adipose tissue was excised at the beginning of surgery, when at least 1 cm^2^ of adipose tissue was retrieved from the edge of the major omentum using diathermia. Cohort 3 was used for *SIK1* mRNA expression analysis by microarray [31]. Cohorts 1 and 2 are described in Tables 1 and 2, respectively.

**Table 1:** Clinical characteristics of cohort 1

	Before weight loss (A)	After weight loss (B)	Non-obese controls (C)	*p* value	
				A vs B	A vs C
Age (years)	44 ± 9	46 ± 9	47 ± 11		0.307
BMI (kg/m^2^)	42.3 ± 4.8	28.3 ± 4.5	24.7 ± 2.2	<0.001	<0.001
Waist circumference (cm)	129 ± 10.8	95.5 ± 12.6	86 ± 7.0	<0.001	<0.001
Waist-to-hip ratio	1.00 ± 0.06	0.92 ± 0.07^a^	0.87 ± 0.06	<0.001	<0.001
P-glucose (mmol/l)	5.7 ± 1.5	4.9 ± 0.6^b^	5.0 ± 0.4	<0.001	0.025
P-insulin (pmol/l)	16.5 ± 9.31	4.63 ± 1.87^c^	3.88 ± 1.57	<0.001	<0.001
P-triacylglycerols (mmol/l)	1.63 ± 0.81^d^	1.01 ± 0.49	0.92 ± 0.63	<0.001	<0.001
Total cholesterol (mmol/l)	4.8 ± 0.9	4.2 ± 0.9	5.0 ± 1.2	<0.001	0.729
HDL-cholesterol (mmol/l)	1.1 ± 0.2	1.5 ± 0.4	1.6 ± 0.4	<0.001	<0.001
LDL-cholesterol (mmol/l)	3.3 ± 0.8^e^	2.5 ± 0.7	3.1 ± 1.0^f^	<0.001	0.112
ApoA-I (g/l)	1.20 ± 0.19	1.42 ± 0.33	1.45 ± 0.23	<0.001	<0.001
ApoB (g/l)	0.95 ± 0.24	0.81 ± 0.21	0.90 ± 0.28	0.001	0.363
Clamp (mmol glucose kg^−1^ body weight min^−1^)	0.0202 ± 0.0066^g^	0.0369 ± 0.0075^b^	0.0439 ± 0.0090	<0.001	<0.001
HOMA-IR	4.30 ± 2.93	1.00 ± 0.43^b^	0.85 ± 0.37^b^	<0.001	<0.001
Subjects (*n*)	37	37	19		

Data represent value ± SD

^a^ *n* = 36; ^b^ *n* = 33; ^c^ *n* = 32; ^d^ *n* = 36; ^e^ *n* = 36; ^f^ *n* = 18; ^g^ *n* = 33

Apo, apolipoprotein; P, fasting plasma

**Table 2:** Characteristics of cohort 2

	Non-obese	Obese	All
Age (years)	54 ± 12.4	43 ± 11.2^a^	45 ± 12.4^b^
BMI (kg/m^2^)	25 ± 3.0	42 ± 9.6	39 ± 11.2
Sex, male (female)	3 (2)	4 (15)	7 (17)
Subjects (*n*)	5	19	24

Data represent value ± SD

^a^ *n* = 15; ^b^ *n* = 20

### Ethics

All participants were given written and oral information about the study before providing their written informed consent. Human studies were approved by the Regional Ethical Review Boards at Karolinska Institutet and Lund University. Animal experiments were approved by the Regional Ethical Committee on Animal Experiments in Malmö/Lund.

### Isolation of primary human adipocytes

Human adipocytes were isolated from subcutaneous or omental (cohort 2) adipose tissue by collagenase digestion. Isolated cells were then lysed and homogenised as described in detail in the ESM Methods.

### Cell culture and treatments

3 T3-L1 fibroblasts were obtained from ATCC (Manassas, VA, USA) and cultured and differentiated to adipocytes [32]. Human mesenchymal stem cells (hMSCs) were isolated from adipose tissue, cultured and differentiated in vitro as described previously [27]. Fully differentiated adipocytes were treated with mouse or human TNF-α (Sigma-Aldrich, St Louis, MO, USA), or a pan-SIK-inhibitor (HG-9-91-01, kindly provided by K. Clark, MRC Protein Phosphorylation Unit, University of Dundee, Dundee, UK [23]), or electroporated with siRNA [33]. In single siRNA treatments, 13 nmol/l of specific and 27 nmol/l of non-targeting siRNA were used to keep the final siRNA concentration at 40 nmol/l. After treatments (concentrations and time points are indicated in figure legends), cells were lysed in QIAzol (Qiagen, Hilden, Germany) for gene expression analysis, or harvested for protein analysis [25]. The siRNA used are listed in the ESM Methods.

### Gene expression analysis

Extraction of total RNA, complementary DNA (cDNA) synthesis and real-time RT-PCR (qRT-PCR) was performed as described in detail in the ESM Methods. The primers and DNA oligos used are listed in the ESM Methods. For *SIK2* and *SIK3* mRNA expression analysis (cohort 1), the samples were numerically coded and all were analysed on the same PCR plate in a randomised order.

### Western blotting and protein analysis

Lysates (5–20 μg protein) were analysed by SDS-PAGE and western blotting [32]. The antibodies used are listed in the ESM Methods. For analysis of SIK2 protein levels in primary human adipocytes (cohort 2), signals were normalised to β-actin [34] as well as to an internal control sample loaded in the outer wells of each gel, and expressed as fold relative to one individual. The samples were run on gels twice, loaded in randomised orders with regards to BMI.

### In vitro kinase assay

Lysates (adipocytes 10–20 μg protein, adipose tissue 100–200 μg protein) were incubated with SIK2 or SIK3 antibodies coupled to protein G-Sepharose (GE Healthcare, Little Chalfont, UK), and phosphotransferase activity towards the peptide substrates HDAC5tide or Sakamototide (200 μmol/l, GL Biochem, Shanghai, China) was measured as described previously [25, 32]. One unit of activity (U) was defined as that which catalysed the transfer of 1 nmol ^32^P/min to the substrate.

### Glucose transport

Basal and insulin-stimulated uptake of [^3^H]-glucose was measured as described previously [27, 33] after siRNA silencing of SIKs for 96 h, or treatment with HG-9-91-01 as indicated in figure legends.

### Immunofluorescence and total internal reflection fluorescence-imaging

Primary adipocytes were isolated from male Sprague Dawley rats as described [32], suspended in KRB-HEPES (10% vol./vol.), pre-treated with HG-9-91-01 and stimulated with insulin for 5 min. Cells were then washed, fixed and stained, and subjected to total internal reflection fluorescence (TIRF)-imaging as described in detail in the ESM Methods.

### Statistical analysis

All values are presented as means (± SD). Data that were not normally distributed were logarithmically (log_10_) transformed. Statistical tests were performed using GraphPad Prism 6 (La Jolla, CA, USA) or SPSS (IBM, Armonk, NY, USA). Details of the statistical tests performed are provided in figure legends.

## Results

### *SIK2* is the dominant SIK in human adipocytes

**Fig. 1:**
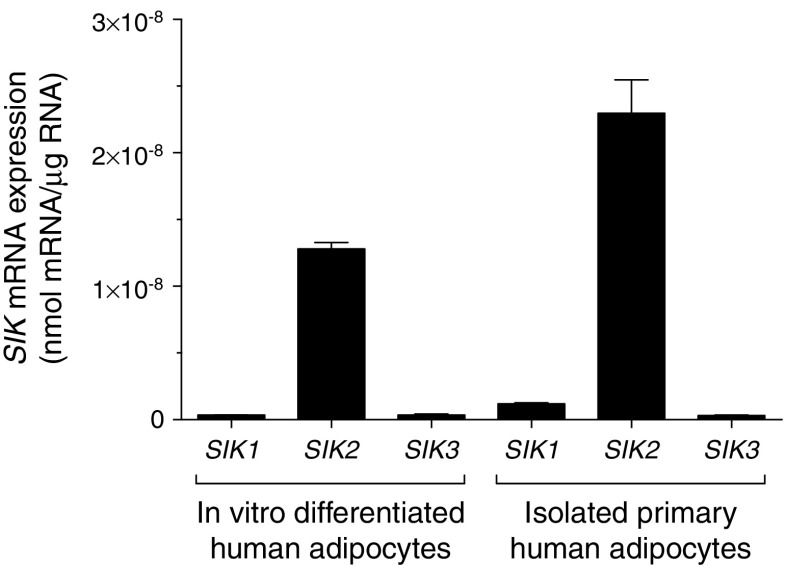
*SIK2* is the dominant SIK in human adipocytes. Absolute levels of *SIK* mRNA in human adipocytes differentiated in vitro (pooled from three passages), and primary human adipocytes isolated from subcutaneous WAT (cohort 2, pooled from two individuals, BMI 29–30 kg/m^2^)

To elucidate expression levels of each SIK isoform in human adipocytes, we performed absolute quantification of *SIK* mRNA transcripts using gene-specific DNA oligos in two different human adipocyte models. As shown in Fig. 1, *SIK2* was clearly the most abundant isoform relative to *SIK1* and *SIK3*. In order to estimate the contribution of each isoform to total SIK activity, we performed in vitro kinase assays of SIK2 and SIK3 in human adipose tissue or adipocyte lysates. This again suggested that SIK2 is the dominant isoform but that the contribution of SIK3 to total SIK activity is higher than anticipated from the mRNA expression analysis (ESM Fig. 1a,b). Due to the lack of specific immunoprecipitating antibodies we were not able to do the corresponding analysis for SIK1.

### Expression of *SIK2* and *SIK3* mRNA is regulated by weight change and correlates negatively to insulin resistance

**Fig. 2:**
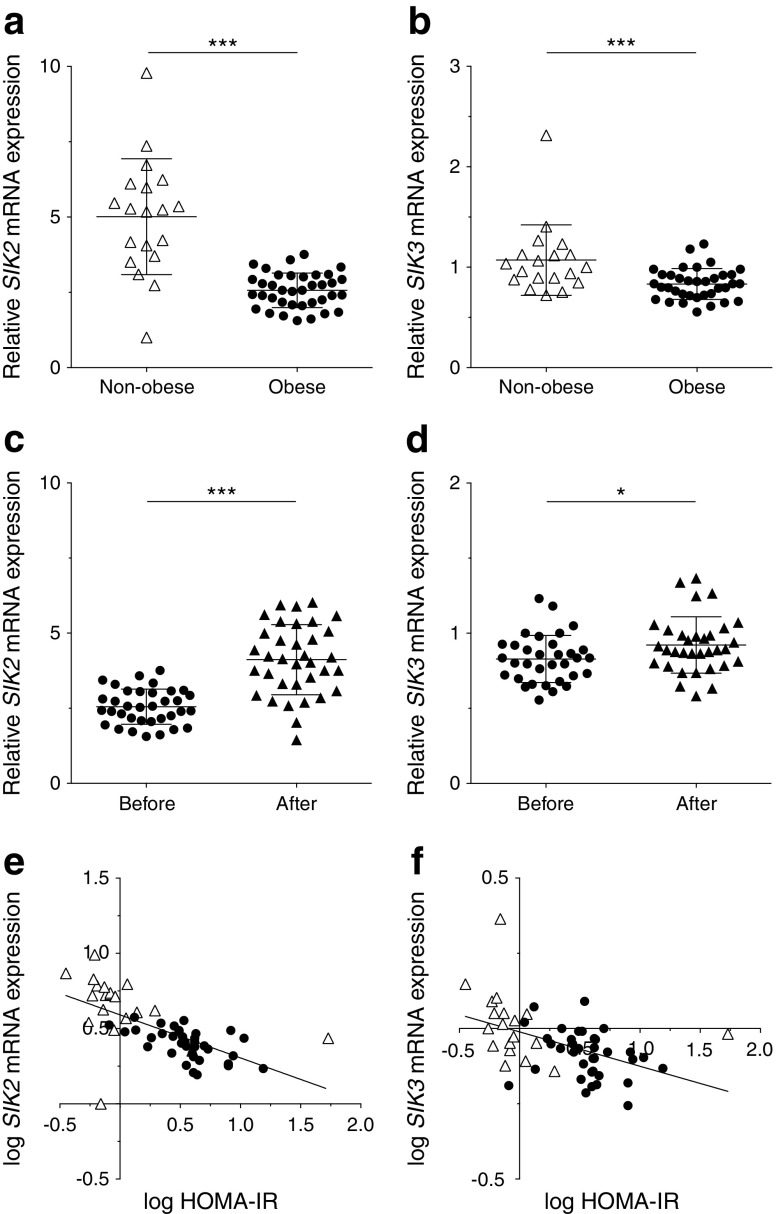
Expression of *SIK2* and *SIK3* mRNA is regulated by weight change and correlates negatively to insulin resistance. Expression of *SIK2* and *SIK3* in subcutaneous WAT (cohort 1, BMI 20–55 kg/m^2^). *LRP10* was used as reference gene and the sample with lowest expression as calibrator. (**a**) *SIK2* and (**b**) *SIK3* mRNA expression in non-obese (white triangles, *n* = 19) and obese (black circles, *n* = 37 and *n* = 36, respectively) individuals. Statistical significance determined by two-tailed unpaired Student’s *t* test. (**c**) *SIK2* (*n* = 35) and (**d**) *SIK3* (*n* = 33) mRNA expression before (black circles) and after weight loss (2 years after bariatric surgery, black triangles) in obese individuals. Statistical significance determined by two-tailed paired Student’s *t* test. (**e**) *SIK2* (*n* = 56) and (**f**) *SIK3* (*n* = 55) mRNA expression plotted against HOMA-IR. Correlations made using Pearson correlation test. One outlier in (**a**, **b**, **e**, **f**) was excluded from statistical analysis due to deviant raw CT values of the reference gene (4.5 SD higher than the average CT value) that is believed to be due to a technical error. *p < 0.05, ***p < 0.001

Considering the proposed roles of SIK2 and SIK3 in the regulation of lipid metabolism [9, 17, 20, 21], we analysed the mRNA expression of these SIK isoforms in subcutaneous WAT from non-obese and obese women (cohort 1). The expression of *SIK2* and *SIK3* was significantly lower in adipose tissue from obese compared with non-obese individuals (Fig. 2a, b). Moreover, *SIK2* and *SIK3* were increased in response to weight loss in previously obese individuals (Fig. 2c, d). Furthermore, *SIK2* displayed a strong negative association with in vivo insulin resistance, measured by HOMA-IR (Fig. 2e; *r* = −0.76, *p* < 0.001). This finding was independent of BMI and age of the individuals (beta coefficient −0.45, *p* = 0.006). *SIK3* was also negatively associated with HOMA-IR (Fig. 2f; *r* = −0.47, *p* = 0.0004) but did not remain so after adjusting for BMI and age. Additionally, we analysed the expression of *SIK1* mRNA in subcutaneous WAT (cohort 3, [31]) and found that *SIK1*, in contrast to *SIK2* and *SIK3,* displayed a higher expression in obese than in non-obese individuals (ESM Fig. 1c).

### SIK2 protein levels and kinase activity are downregulated in human obesity

**Fig. 3:**
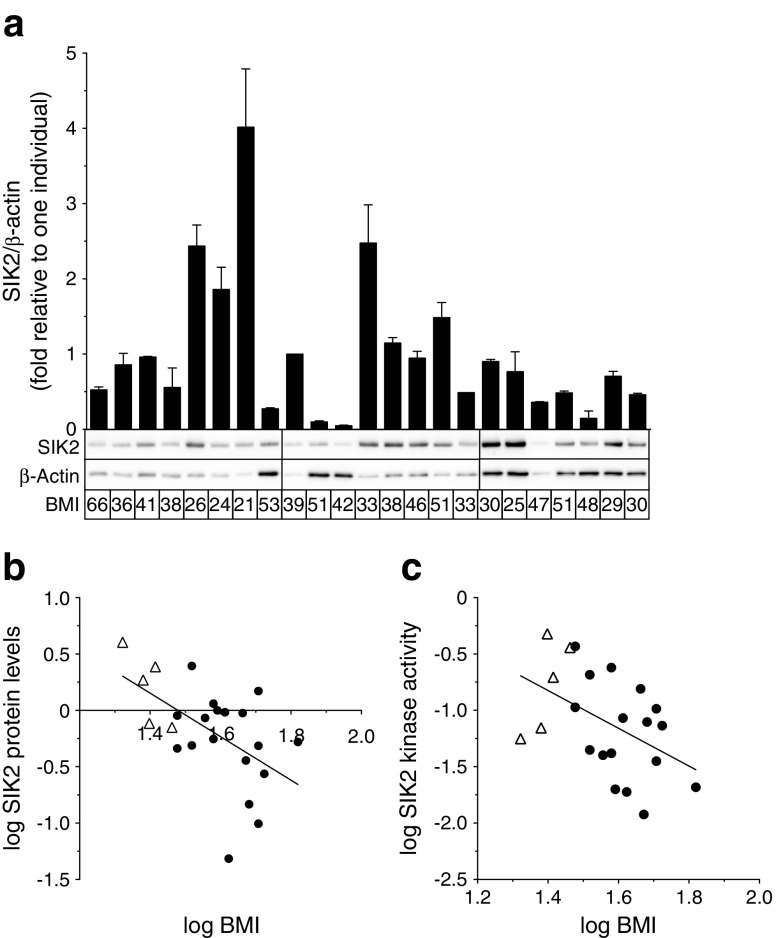
SIK2 protein levels and kinase activity are downregulated in human obesity. SIK2 protein levels determined in (**a**), and kinase activity in subcutaneous adipocytes from non-obese (white triangles) and obese (black circles) individuals (cohort 2, BMI 21–66 kg/m^2^). (**a**) SIK2 protein levels. β-actin was used as loading control and its expression did not change with BMI (ESM Fig. 2f). Graph shows means + SD from two independent gel runs. Each bar represents one individual and representative blots are shown below the graph (*n* = 23). (**b**) SIK2 protein levels plotted against BMI (*n* = 23). (**c**) SIK2 in vitro kinase activity against the peptide substrate HDAC5tide plotted against BMI (*n* = 22). Correlations made using Pearson correlation test

Based on the observation that *SIK2* was most distinctly affected in human obesity, we chose to mainly focus our further studies on this isoform. SIK2 protein levels were determined by western blotting in primary human adipocytes (cohort 2, Fig. 3a). As shown in Fig. 3b, SIK2 displayed a negative correlation to BMI in subcutaneous adipocytes (*r* = −0.55, *p* = 0.006). Similarly, SIK2 kinase activity was negatively correlated to BMI (Fig. 3c; *r* = −0.47, *p* = 0.03). Although we observed a strong positive correlation between SIK2 kinase activity and protein levels (ESM Fig. 2a; *r* = 0.98, *p* < 0.001), the kinase activity in relation to the amount of SIK2 protein (specific activity) was also negatively correlated to BMI (ESM Fig. 2b; *r* = −0.52, *p* = 0.01). There was no significant association between SIK2 and BMI in omental adipocytes (ESM Fig. 2c). However, paired analysis of subcutaneous and omental adipocytes from the same individual revealed that SIK2 protein levels in these depots were similar (ESM Fig. 2d) and co-varied (ESM Fig. 2e; *r* = 0.72, *p* = 0.006).

### SIK2 and SIK3 are downregulated by TNF-α in adipocytes

**Fig. 4:**
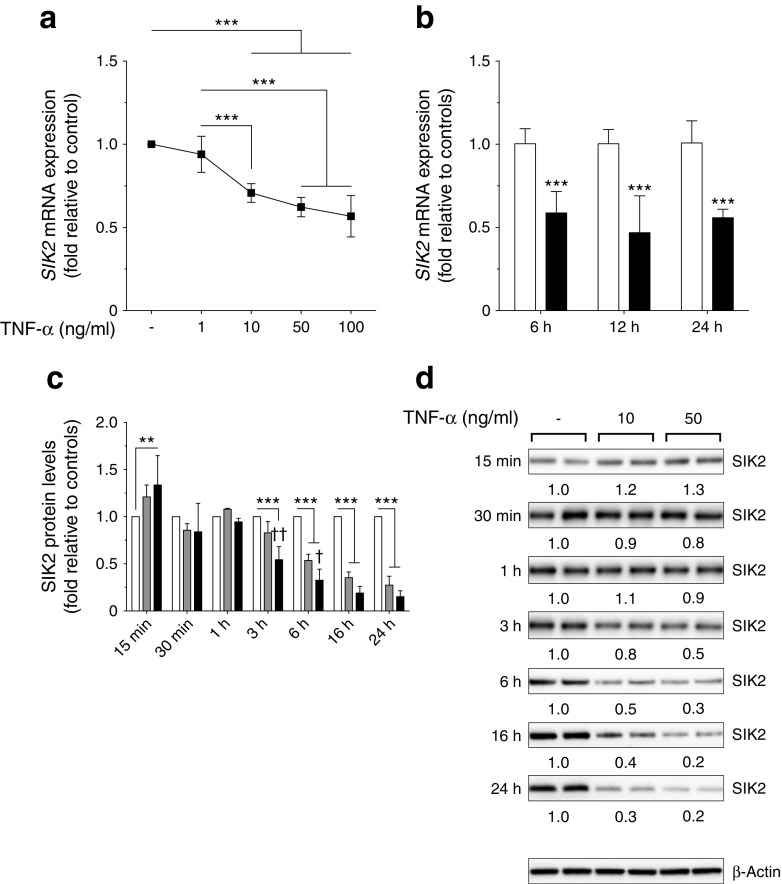
SIK2 is downregulated by TNF-α in adipocytes. *SIK2* mRNA expression in human adipocytes differentiated in vitro, treated with (**a**) increasing concentrations of TNF-α for 24 h and (**b**) TNF-α 50 ng/ml (black bars) or vehicle (white bars) for indicated time points (*n* = 3 independent experiments performed in duplicate). 18S rRNA was used as reference gene. (**c**) SIK2 protein levels in 3T3-L1 adipocytes treated with TNF-α 10 ng/ml (grey bars) or 50 ng/ml (black bars), or vehicle (white bars) for indicated time points (*n* = 3 independent experiments). (**d**) Representative blots with mean values for relative protein levels below. β-Actin and heat shock protein 90 (HSP90) were used as loading controls. Statistical significance determined by two-way ANOVA with Tukey’s (**a**, **c**) or Sidak’s (**b**) multiple comparisons post hoc tests. **p < 0.01, ***p < 0.001, and ^†^ *p* < 0.05, ^††^ *p* < 0.01 TNF-α 10 ng/ml vs 50 ng/ml

To investigate whether the inflammation often associated with obesity and insulin resistance [35, 36] influences the expression of SIK isoforms, adipocytes were treated with TNF-α. As shown in Fig. 4a, the expression of *SIK2* mRNA decreased in a dose-dependent manner in human adipocytes, and was reduced by 40% after 6 h (Fig. 4b). Moreover, SIK2 protein displayed a marked dose- and time-dependent reduction in 3T3-L1 adipocytes (Fig. 4c,d). Similar to SIK2, the protein levels of SIK3 were also reduced although at a slower rate (ESM Fig. 3a, b). Furthermore, acute treatment (<3 h) with TNF-α did not change the kinase activity (ESM Fig. 3c) or the specific phosphorylation of SIK2 on Ser343, Ser358 or Thr484 (ESM Fig. 3d–f). The phosphorylation of mitogen-activated protein kinase (MAPK) p38, a target for TNF-α signalling [37], was analysed to confirm the activity of TNF-α, and the expression of the adipocyte marker adipose triacylglycerol lipase (ATGL) was analysed to exclude any non-specific effects of the treatment (ESM Fig. 3g).

### SIKs promote glucose uptake in human adipocytes and influence insulin signalling and GLUT4 localisation

**Fig. 5:**
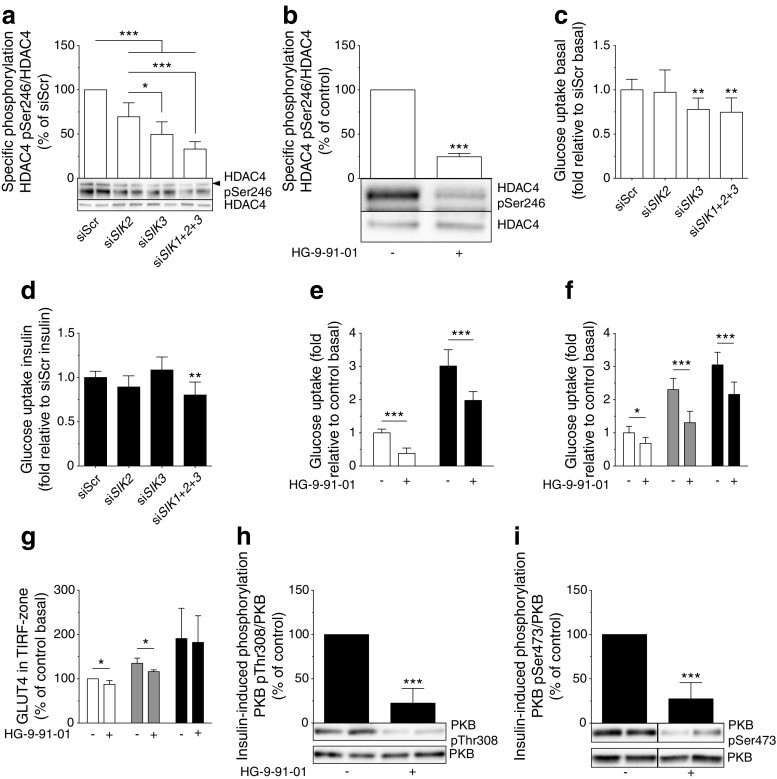
SIKs promote glucose uptake in human adipocytes and influence insulin signalling and GLUT4 localisation. SIK isoforms were silenced using siRNA or inhibited with HG-9-91-01 in adipocytes. Phosphorylation of HDAC4 at Ser246, normalised to total HDAC4, in human adipocytes differentiated in vitro, treated with (**a**) *SIK* siRNA (*n* = 4–8 independent experiments) or (**b**) HG-9-91-01 (3 μmol/l, overnight) (*n* = 3 independent experiments). Glucose uptake at basal (white bars) and after insulin stimulation (10 nmol/l, black bars) in human adipocytes differentiated in vitro, treated with (**c**, **d**) *SIK* siRNA (*n* = 4 independent experiments performed in triplicate) or (**e**) HG-9-91-01 (3 μmol/l, overnight) (*n* = 3 independent experiments performed in triplicate). To visualise differences and facilitate statistical analysis, basal glucose uptake (normalised to siScr-treated cells) and insulin-stimulated glucose uptake (normalised to cells treated with siScr and insulin) are presented in (**c**) and (**d**), respectively. The stimulation index in these experiments was about tenfold. (**f**) Glucose uptake at basal (white bars) and after insulin stimulation (1 nmol/l, grey bars and 10 nmol/l, black bars) in human adipocytes differentiated in vitro, treated with HG-9-91-01 (10 μmol/l, 1 h) (*n* = 4 independent experiments performed in triplicate). (**g**) Presence of GLUT4 in the TIRF-zone at basal (white bars) and after insulin stimulation (0.1 nmol/l, grey bars and 1 nmol/l, black bars) in primary rat adipocytes treated with HG-9-91-01 (10 μmol/l, 1 h), measured by immunofluorescence TIRF microscopy (*n* = 3–5 independent experiments each in which 30–50 cells per condition were analysed). Insulin-induced phosphorylation of PKB/Akt at Thr308 (**h**) and Ser473 (**i**) normalised to total PKB/Akt, in human adipocytes differentiated in vitro, treated with HG-9-91-01 (3 μmol/l, overnight) followed by insulin (10 nmol/l, 15 min) (*n* = 4 independent experiments). Statistical significance determined by one-way ANOVA with Tukey’s (**a**) or Dunnett’s (**c**, **d**) multiple comparisons post hoc tests, two-way ANOVA with Sidak’s (**e**–**g**) multiple comparisons post hoc test, and unpaired Student’s *t* test (**b**, **h**, **i**). *p < 0.05, **p < 0.01, ***p < 0.001

Since SIK2 has been shown to promote glucose uptake in rodent adipocytes [19, 20], we investigated the impact of reduced expression/activity of SIK isoforms on this process in human adipocytes. We silenced all three SIK isoforms individually or simultaneously, as well as employed the highly selective pan-SIK-inhibitor HG-9-91-01 [23]. The level of *SIK1* mRNA was very low compared with *SIK2* (data not shown) and was only reduced to a modest degree by siRNA (si*SIK1*, ESM Fig. 4a). At protein level, there was no effect of si*SIK1* (ESM Fig. 4b) and this condition was thus excluded in further analyses. Treatment with siRNA against SIK2 (si*SIK2*) or SIK3 (si*SIK3*) caused efficient silencing of mRNA and protein expression (ESM Fig. 4c–f). Silencing of SIK3 was accompanied by a slight increase in *SIK2* mRNA (ESM Fig. 4c) but SIK2 protein was unchanged (ESM Fig. 4d). However, silencing of SIK2 was associated with compensatory upregulation of both SIK1 and SIK3 protein levels (ESM Fig. 4b, f). The presence of residual SIK activity when silencing SIK2 was demonstrated by a modest reduction in p-HDAC4, an SIK substrate whose phosphorylation we used as a readout for total SIK activity (Fig. 5a). Silencing of SIK3 resulted in a larger reduction in p-HDAC4, and simultaneous silencing of SIK1–3 (si*SIK1+2+3*, Fig. 5a) or pan-SIK inhibition (Fig. 5b) reduced p-HDAC4 even further. Neither basal (Fig. 5c) nor insulin-stimulated (Fig. 5d) glucose uptake was changed in si*SIK2*-treated adipocytes compared with controls. Silencing of SIK3 was associated with lower basal glucose uptake (Fig. 5c) and si*SIK1+2+3*, or overnight SIK inhibition, resulted in significantly lower glucose uptake both in the absence and presence of insulin (Fig. 5c–e).

To get insight into mechanisms underlying the effect of SIKs on glucose uptake we monitored the expression of glucose transporters. We observed an increase in GLUT4 protein levels following silencing of SIK3 or all three isoforms simultaneously (ESM Fig. 5a) and si*SIK2* induced GLUT1 (ESM Fig. 5b). Based on the observation that the positive effect of SIKs on glucose uptake is not likely to be mediated by changes in GLUT1 and GLUT4 levels we investigated the hypothesis that SIKs promote glucose uptake by directly stimulating insulin signalling and/or GLUT4 localisation. Consequently, we treated adipocytes with HG-9-91-01 for 1 h and analysed glucose uptake. Similar to overnight treatment (Fig. 5e), 1 h of SIK inhibition was sufficient to lower both basal glucose uptake and uptake stimulated by sub-maximal (1 nmol/l) and maximal (10 nmol/l) doses of insulin in human adipocytes (Fig. 5f). Correspondingly, the amount of GLUT4 close to the plasma membrane was reduced in rat adipocytes, both in the basal state and in the presence of sub-maximal insulin (0.1 nmol/l) (Fig. 5g). Furthermore, pan-SIK inhibition in human adipocytes was associated with a marked reduction of the insulin-induced phosphorylation of protein kinase B (PKB/Akt), a key regulator of GLUT4 translocation [38, 39], at both Thr308 (Fig. 5h) and Ser473 (Fig. 5i).

## Discussion

This study is the first to demonstrate SIK expression and function in human adipose tissue. We have shown that *SIK2* and *SIK3* are markedly downregulated in adipose tissue from obese or insulin-resistant humans (independently of BMI or age for *SIK2*) and that the expression is regulated in response to weight change and inflammation (TNF-α). Moreover, SIKs promote insulin signalling, GLUT4 translocation to the plasma membrane and glucose uptake in adipocytes.

Our study demonstrates that interspecies differences exist in the regulation of SIK2 expression and activity in WAT. In contrast to what was previously reported in obese mice [9], we found that SIK2 expression (mRNA and protein) and activity in adipose tissue and adipocytes were downregulated in human obesity. As adipose tissue inflammation is a key feature of obesity and insulin resistance [35, 36], we hypothesised that SIK expression is regulated by the inflammatory cytokine TNF-α. Indeed, both SIK2 and SIK3 were rapidly downregulated by TNF-α. The molecular mechanisms mediating transcriptional regulation of SIK2 and SIK3 remain to be elucidated. However, the rapid decrease in gene and protein expression indicates that the effect of TNF-α is probably direct, and not secondary to TNF-induced changes in the expression of other genes. Since *SIK2* and *SIK3* expression was lower in insulin-resistant individuals it is also possible that a functional insulin response is needed for SIK transcription.

A critical question is how the reduced SIK2 and SIK3 expression in obesity impacts adipose tissue physiology. Previous studies in rodents have proposed that SIKs regulate glucose uptake in adipocytes [19, 20]. In our study, we demonstrate an important role for SIK isoforms in promoting basal and insulin-stimulated glucose uptake also in human adipocytes, using both genetic (siRNA) and pharmacological (pan-SIK inhibition) approaches. Silencing of SIK2 induced compensatory upregulation of SIK1 and SIK3, leading to only a marginal reduction in total SIK activity, making it difficult to conclude on the individual role of SIK2. Given the low abundance of *SIK3* relative to *SIK2* in human adipocytes, at first we anticipated that the functional contribution of SIK3 would be much smaller than that of SIK2. However, kinase activity measurements and si*SIK3* treatment revealed that there is a significant level of SIK3 activity in human adipose tissue that contributes to the positive effect of SIKs on glucose uptake. We were not able to achieve silencing of SIK1 at protein level, making any conclusions about the role of SIK1 uncertain.

When exploring mechanisms for the regulation of glucose uptake by SIKs we noted that, in contrast to murine adipocytes [19, 20], SIK isoforms do not promote GLUT expression in human adipocytes. Thus, our data suggest that the positive effect of SIKs on glucose uptake in human adipocytes is not likely to be mediated by altered protein levels of glucose transporters. However, the rate of glucose uptake is ultimately dependent on the number of transporters present on the cell surface and not the overall cellular levels. Accordingly, our data suggest that SIKs promote plasma membrane localisation of GLUT4 in adipocytes. The upstream mechanism involves positive effects of SIKs on the insulin-induced phosphorylation and activation of PKB/Akt. The fact that the effect of SIK inhibition on GLUT4 localisation was smaller than that on glucose uptake and detected only at a sub-maximal dose of insulin could be a result of methodological (snapshot vs cumulative assay) and species (rat vs human) differences. Moreover, since GLUT4 translocation and glucose uptake were also reduced by SIK inhibition in the basal state we do not rule out the possibility that additional mechanisms may contribute. The fact that the ability of insulin to induce these processes (stimulation index, fold change) was not altered even though the phosphorylation of PKB/Akt was markedly blunted indicates that the effect of SIKs on basal glucose uptake and GLUT4 localisation is probably mediated by a distinct, yet unknown, mechanism.

An important question to answer in future studies is if the differential expression of SIKs in adipose tissue plays a causal role in the development of obesity or insulin resistance in vivo. Mice with global deficiency of SIK2 displayed no weight phenotype [20], arguing against a causal relationship between SIK2 downregulation and obesity. However, it is quite possible that some effects of SIK2 deficiency are masked by compensatory mechanisms due to embryonic loss of the protein or isoform redundancy. Although reduced PKB/Akt activation might not fully explain the effects we observed on GLUT4 translocation and glucose uptake in cells, these results per se, as well as the strong negative association of *SIK2* expression with HOMA-IR, suggest that downregulation of *SIK2* and *SIK3* in obesity might contribute to the development of insulin resistance in vivo—at least in adipose tissue. In line with this, *Sik2* ^-/-^ mice showed some degree of insulin resistance in their adipose tissue [20]. Considering the low expression level of *SIK1* in human adipose tissue, we are not sure of the physiological relevance of the differential expression of this isoform in obesity. Previous studies have demonstrated that *Sik1* is upregulated in skeletal muscle, liver and adipose tissue of obese mice [9, 40] and this has been linked to the development of insulin resistance [40]. Given the compensatory upregulation of SIK1 that we observed when silencing SIK2 in adipocytes, it is possible that increased *SIK1* expression in adipose tissue from obese individuals is secondary to downregulation of *SIK2* in these individuals.

In summary, we have demonstrated that *SIK2* and *SIK3* are downregulated in human obesity and insulin resistance. Furthermore, SIKs promote glucose uptake in human adipocytes, at least partly through direct mechanisms. In future studies it will be important to identify molecular targets of SIK2 and SIK3 that could be involved in the regulation of PKB/Akt phosphorylation and GLUT trafficking.

## Electronic supplementary material

Below is the link to the electronic supplementary material.

ESM (PDF 6135 kb)

## Abbreviations

AMPK: AMP-activated protein kinase
CRTC: CREB-regulated transcription coactivator
HDAC: Histone deacetylase
LKB1: Liver kinase B1
PKA: Protein kinase A
PKB/Akt: Protein kinase B
SIK: Salt-inducible kinase
siRNA: Small interfering RNA
TIRF: Total internal reflection fluorescence
WAT: White adipose tissue
