# Supplementary material for: Salt-inducible kinase 2 and -3 are downregulated in adipose tissue from obese or insulin-resistant individuals: implications for insulin signalling and glucose uptake in human adipocytes
Source: Diabetologia. 2016 Nov 2;60(2):314–23. doi: 10.1007/s00125-016-4141-y (PMC6518086; doi:10.1007/s00125-016-4141-y)
Supplement: Supplementary file 1 — (PDF 6135 kb) [file 125_2016_4141_MOESM1_ESM.pdf]

ESM Methods

Isolation of primary human adipocytes

Human adipocytes were isolated from adipose tissue (cohort 2) by collagenase (1 mg/ml) digestion in a shaking incubator at 37°C. Digests were filtered and washed with KRB-HEPES containing 25 mmol/l HEPES (pH 7.4), 2 mmol/l glucose, 1 % (wt./vol.) BSA and 200 nmol/l adenosine. For gene expression analysis, isolated cells were washed with cold PBS and homogenized using a 0.8 mm needle in QIAzol (Qiagen, Hilden, Germany). For protein analysis, isolated cells were washed with KRB-HEPES (without BSA) and homogenised using a 0.4 mm needle in 50 mmol/l TRIS-HCl (pH 7.5), 0.27 mol/l sucrose, 1 mmol/l EDTA, 1 mmol/l EGTA, 5 mmol/l sodium pyrophosphate, 1 mmol/l sodium orthovanadate, 50 mmol/l sodium fluoride, 1 mmol/l dithiothreitol and complete protease inhibitor cocktail (one tablet/50 ml, Roche, Mannheim, Germany). Homogenates were centrifuged at 100 g for 5 min (4°C), the fat was removed and the infranatant solubilised by adding 1 % (wt./vol.) NP-40. After incubation on ice for 15 min, lysates were centrifuged at 13 000 g for 15 min (4°C) and the supernatants were collected. Protein concentration was determined by the Bradford assay.

siRNA

The following siRNA were used for electroporation: Dharmacon siGENOME SMARTpool for human *SIK1* (M-003959-05), human *SIK2* (M-004778-03), human *SIK3* (M-004779-03) and non-targeting control (D-001206-13) were from GE Healthcare.

Quantitative real-time RT-PCR

Total RNA was extracted from human adipocytes or adipose tissue using RNeasy Lipid Tissue Mini Kit (Qiagen) according to the manufacturer's instructions. For absolute quantification of SIK mRNA, total RNA (1 μg) was treated with DNase I (Amplification Grade, Invitrogen, Life Technologies, Carlsbad, CA, USA) and reverse transcribed to cDNA with SuperScript II RT (Invitrogen) using dNTP mix (Invitrogen) and random hexamers (Applied Biosystems, Life Technologies) according to the manufacturer's instructions. Custom-designed DNA oligos were resuspended in RNase-free water and serially diluted to standard curves. Quantitative PCR was performed using Universal PCR Master Mix (Applied Biosystems) with TaqMan Gene Expression Assays, and cDNA (5 ng/reaction) or DNA oligos on a 7900HT Fast Real-Time PCR System (Applied Biosystems). Absolute levels of mRNA were calculated using the standard curve generated from C_T-values of the DNA oligos. For mRNA expression analysis, cDNA synthesis was performed on 0.5 μg of total RNA using the Omniscript RT kit (Qiagen) and random hexamer primers (Invitrogen) according to the manufacturer's instructions. Quantitative real-time PCR was performed using an iCycler IQ (Bio-Rad). Relative gene expression was calculated using the $2^{-\Delta\Delta C_T}$ method [1].

Primers and DNA oligos

The following primers were used for absolute quantification of mRNA transcripts: TaqMan Gene Expression Assays for human *SIK1* (Hs00545020_m1), *SIK2* (Hs01568566_m1) and *SIK3* (Hs00228549_m1) were from Applied Biosystems.

The following primers were used for all other qRT-PCR experiments: QuantiTect Primer Assays for human *SIK2* (QT00034699) and human *SIK3* (QT01669388) were

from Qiagen, and primers 5’-CAGGTTATGGAAACAAAGGAC-3’ (sense) and 5’-GGATTTGCCAGAACTTCTTC-3’ (antisense) for human *SIK1* were from Sigma. Primers for reference genes were: 5’-GATGGAGGCTGAGATTGTG-3’ (sense) and 5’-GAGTCATATCCTGGCGTAAG-3’ (antisense) for *LRP10*, or 5’-TGACTCAACACGGGAAACC-3’ (sense) and 5’-TCGCTCCACCAACTAAGAAC-3’ (antisense) for *18S rRNA* were both from Sigma.

Custom designed DNA oligos for human *SIK1* (sequence 5’-TTC AGC TGA AGC TTC TGA ACC ATC CAC ACA TCA TAA AGC TTT ACC AGG TTA TGG AAA CAA AGG ACA TGC TTT ACA TCG TCA CTG AAT TTG CTA AAA A-3’, 30744 g/mol) and human *SIK2* (sequence 5’-GCG GCT CCT CAG CTC CAG GAC CTT GCT AGC AGC TGC CCT CAG GAA GAA GTT TCT CAG CAG CAG GAA AGC GTC TCC ACT CTC CCT GCC AGC GTG CAT CCC C-3’, 30560 g/mol) were produced by Invitrogen, and human *SIK3* (sequence 5’-GGA TAC ATC CCT ATG GTC ATC AGC CAA CTG-3’, 37298 g/mol) was produced by Biolegio (Nijmegen, The Netherlands).

<u>Antibodies</u>

The following primary antibodies were used for western blotting: rabbit anti-ATGL (Cell Signaling Technology, Danvers, MA, USA, dilution 1:1000), mouse anti-β-actin (Sigma-Aldrich, dilution 1:5000), mouse anti-glyceraldehyde 3-phosphate dehydrogenase (GAPDH) (Sigma-Aldrich, dilution 1:2000), rabbit anti-HDAC4 (Abcam, Cambridge, UK, dilution 1:1000), rabbit anti-p-HDAC4/5/7 Ser246/Ser259/Ser155 (Cell Signaling Technology, dilution 1:1000), mouse anti-HSP90 (BD Biosciences, San Jose, CA, USA, dilution 1:1000), rabbit anti-p-MAPK p38 Thr180/Tyr182 (Cell Signaling Technology, dilution 1:1000), rabbit anti-PKB/Akt (pan) (CST, dilution 1:1000), rabbit anti-p-PKB/Akt Thr308 (Cell Signaling

Technology, dilution 1:1000) and Ser473 (Thermo Fisher Scientific, Waltham, MA, USA, 1:5000) and rabbit anti-SIK1 (Santa Cruz Biotechnologies, Dallas, TX, USA, dilution 1:500). The following antibodies were raised in rabbit and affinity-purified by Innovagen (Lund, Sweden): p-CRTC2 Ser275 (dilution 1 μg/ml) [2], SIK2 (dilution 0.5 μg/ml), p-SIK2 Ser358 (dilution 1 μg/ml) [3], p-SIK2 Thr484 (residues 477-490 of human SIK2, RSGQRRHpTLSEVTN, dilution 1 μg/ml), and SIK3 (dilution 1 μg/ml) [4]. In house antibodies were validated using relevant recombinant wild-type and mutant proteins, as well as material in which kinases were silenced or genetically deleted. Antibody towards p-SIK2 Ser343 (dilution 1 μg/ml) was a generous gift from K. Sakamoto (Diabetes & Circadian Rhythms, Nestlé Institute of Health Sciences, Lausanne, Switzerland) [5]. The GLUT1 antibody (dilution 1:2000) was a generous gift from S.W. Cushman (National Institute of Diabetes and Digestive and Kidney Diseases, National Institutes of Health, Bethesda, USA) and the GLUT4 antibody (dilution 1:5000) was raised in rabbit and purified as described previously [6].

The following secondary antibodies were used: anti-mouse and anti-rabbit horseradish peroxidase (HRP)-conjugated secondary antibodies were from Pierce Biotechnology (Thermo Fischer Scientific, Waltham, MA, USA) and GE Healthcare, respectively.

Immunofluorescence

Cells were washed with KRB-HEPES without BSA, followed by fixation in 4% paraformaldehyde for 6 min, washed in PBS, followed by blocking and permeabilisation in KRB-HEPES with 0.1% Saponin for 30 min. Cells were incubated with primary GLUT4 antibody (dilution 1:1000, [11]) for one hour, washed in PBS with 0.1% Saponin and resuspended in the same buffer containing Alexa Fluor 568 goat anti-mouse IgG secondary antibody (dilution 1:300, Invitrogen, Life

Technologies) and incubated for one hour. Finally, cells were washed in PBS. All steps were performed at room temperature. Imaging of GLUT4 was performed using a Nikon Ti-E eclipse microscope with a 100x Apo TIRF DIC oil immersion objective with a NA of 1.49 (Nikon Instruments, Tokyo, Japan), an iXon Ultra DU-897 EMCCD camera (Andor Technology, Belfast, UK) and 561 laser line (Coherent, Santa Clara, CA, USA) with corresponding filter set. The TIRF-zone depth was set to approximately 65 nm into the specimen. All images were identically subjected to background subtraction. Mean intensity values were obtained from NIS-elements, version: 4.30.02 (Laboratory Imaging, Prague, Czech Republic).

References

[1] Livak KJ, Schmittgen TD (2001) Analysis of relative gene expression data using real-time quantitative PCR and the 2(-Delta Delta C(T)) Method. Methods 25: 402-408

[2] Henriksson E, Sall J, Gormand A, et al. (2015) Salt-inducible kinase 2 regulates CRTCs, HDAC4 and glucose uptake in adipocytes. Journal of cell science 128: 472-486

[3] Henriksson E, Jones HA, Patel K, et al. (2012) The AMPK-related kinase SIK2 is regulated by cAMP via phosphorylation at Ser358 in adipocytes. The Biochemical journal 444: 503-514

[4] Berggreen C, Henriksson E, Jones HA, Morrice N, Goransson O (2012) cAMP-elevation mediated by beta-adrenergic stimulation inhibits salt-inducible kinase (SIK) 3 activity in adipocytes. Cellular signalling 24: 1863-1871

[5] Patel K, Foretz M, Marion A, et al. (2014) The LKB1-salt-inducible kinase pathway functions as a key gluconeogenic suppressor in the liver. Nature communications 5: 4535

[6] Satoh S, Nishimura H, Clark AE, et al. (1993) Use of bismannose photolabel to elucidate insulin-regulated GLUT4 subcellular trafficking kinetics in rat adipose cells. Evidence that exocytosis is a critical site of hormone action. The Journal of biological chemistry 268: 17820-17829

ESM Figure 1

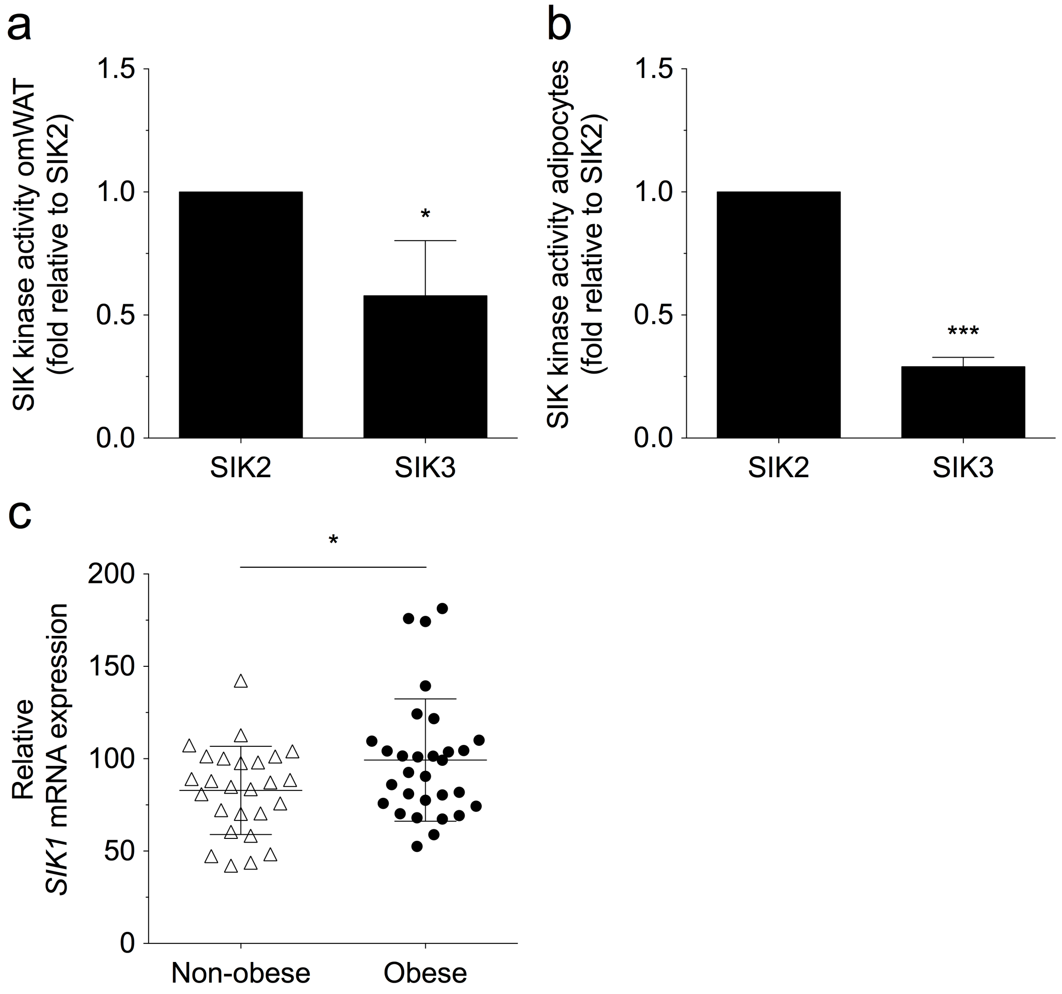

ESM Figure 1. ***Kinase activity of SIK2 and SIK3, and SIK1 mRNA expression in human adipose tissue.*** SIK2 and SIK3 *in vitro* kinase activity against the peptide substrate Sakamototide (encompassing Ser171 in CRTC2) in (**a**) omental adipose tissue (omWAT, cohort 2, age-matched individuals, BMI 21-28 kg/m^2, *n*=3) and (**b**) *in vitro* differentiated human adipocytes (*n*=4). Absolute SIK2 activity ranged from 0.6-1.3 mU/mg in adipose tissue and 46-68 mU/mg in adipocytes. (**c**) Expression of *SIK1* mRNA in subcutaneous WAT from non-obese (white triangles, *n*=26) and obese (black circles, *n*=30) individuals (cohort 3) determined by microarray analysis as described in [1]. Statistical significance determined by two-tailed unpaired Student's t-test. *p<0.05, **p<0.01, ***p<0.001

Reference

[1] Arner E, Mejhert N, Kulyte A, et al. (2012) Adipose tissue microRNAs as regulators of CCL2 production in human obesity. Diabetes 61: 1986-1993

ESM Figure 2

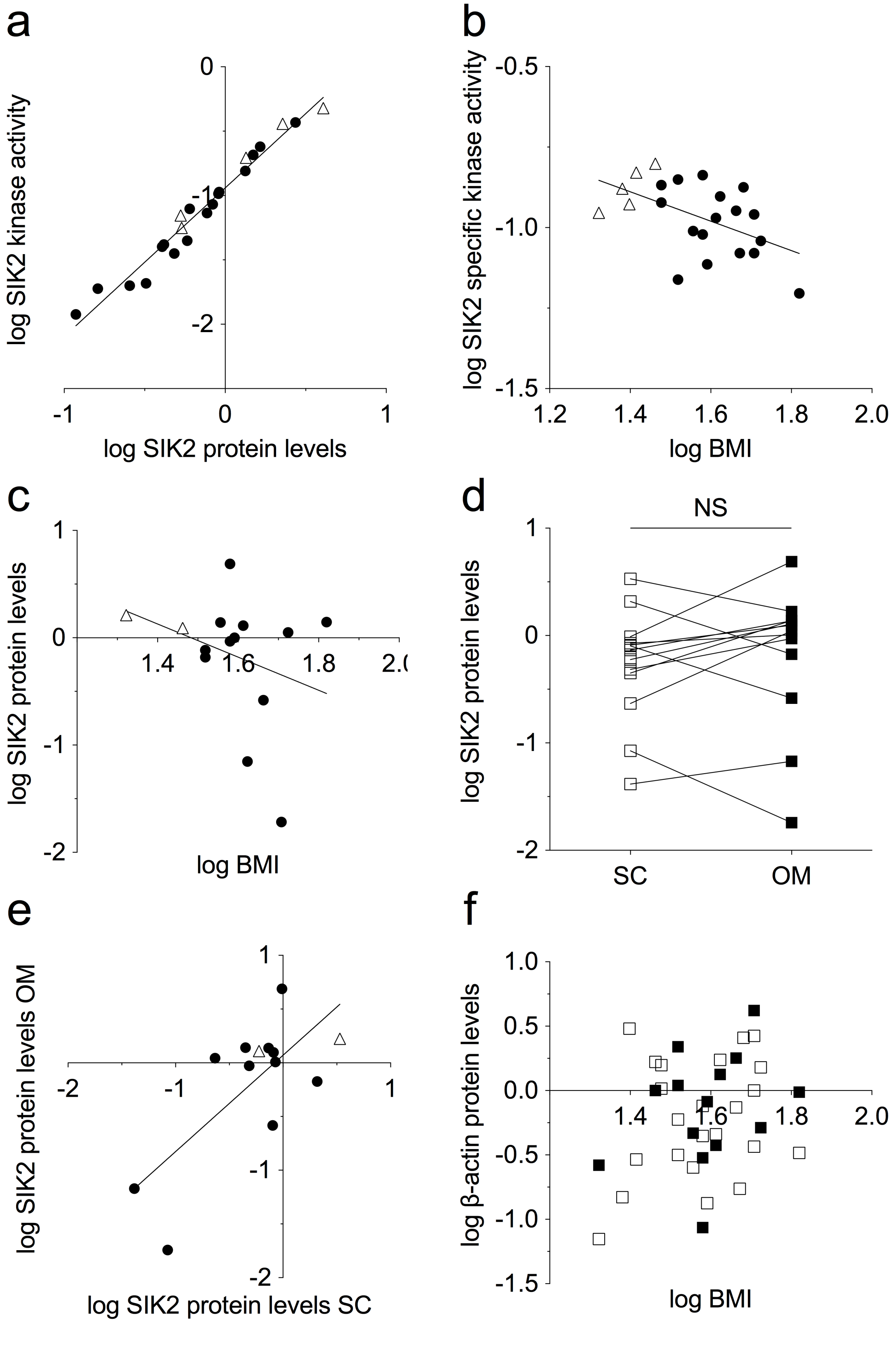

ESM Figure 2. ***Protein levels and kinase activity of SIK2, and protein levels of β-actin, in human subcutaneous and omental adipocytes.*** SIK2 protein levels and

kinase activity, and β-actin protein levels, in primary subcutaneous (SC, white squares) and omental (OM, black squares) adipocytes from non-obese (white triangles) and obese (black circles) individuals (cohort 2, BMI 21-66 kg/m^2). (**a**) SIK2 *in vitro* kinase activity against the peptide substrate HDAC5tide plotted against SIK2 protein levels in SC adipocytes (r=0.98, p<0.001, n=22). (**b**) SIK2 *in vitro* kinase activity against the peptide substrate HDAC5tide in SC adipocytes normalised to SIK2 protein levels determined by western blotting (MS Fig. 3), plotted against BMI (r=-0.52, p=0.01, n=22). (**c**) SIK2 protein levels in OM adipocytes plotted against BMI (r=-0.31, p=0.29, n=14). (**d**) SIK2 protein levels in SC and OM adipocytes from paired samples (p=0.44, n=13). (**e**) SIK2 protein levels in OM adipocytes plotted against SC adipocytes (r=0.72, p=0.006, n=13). Each point represents paired samples from one individual. (**f**) β-actin protein levels in SC and OM adipocytes plotted against BMI (SC, r=0.23, p=0.29, n=23; OM, r=0.30, p=0.30, n=14). Correlations made using Pearson correlation test (**a–c, e–f**) and statistical significance determined by two-tailed paired Student's t-test (**d**). *NS*, non-significant.

ESM Figure 3

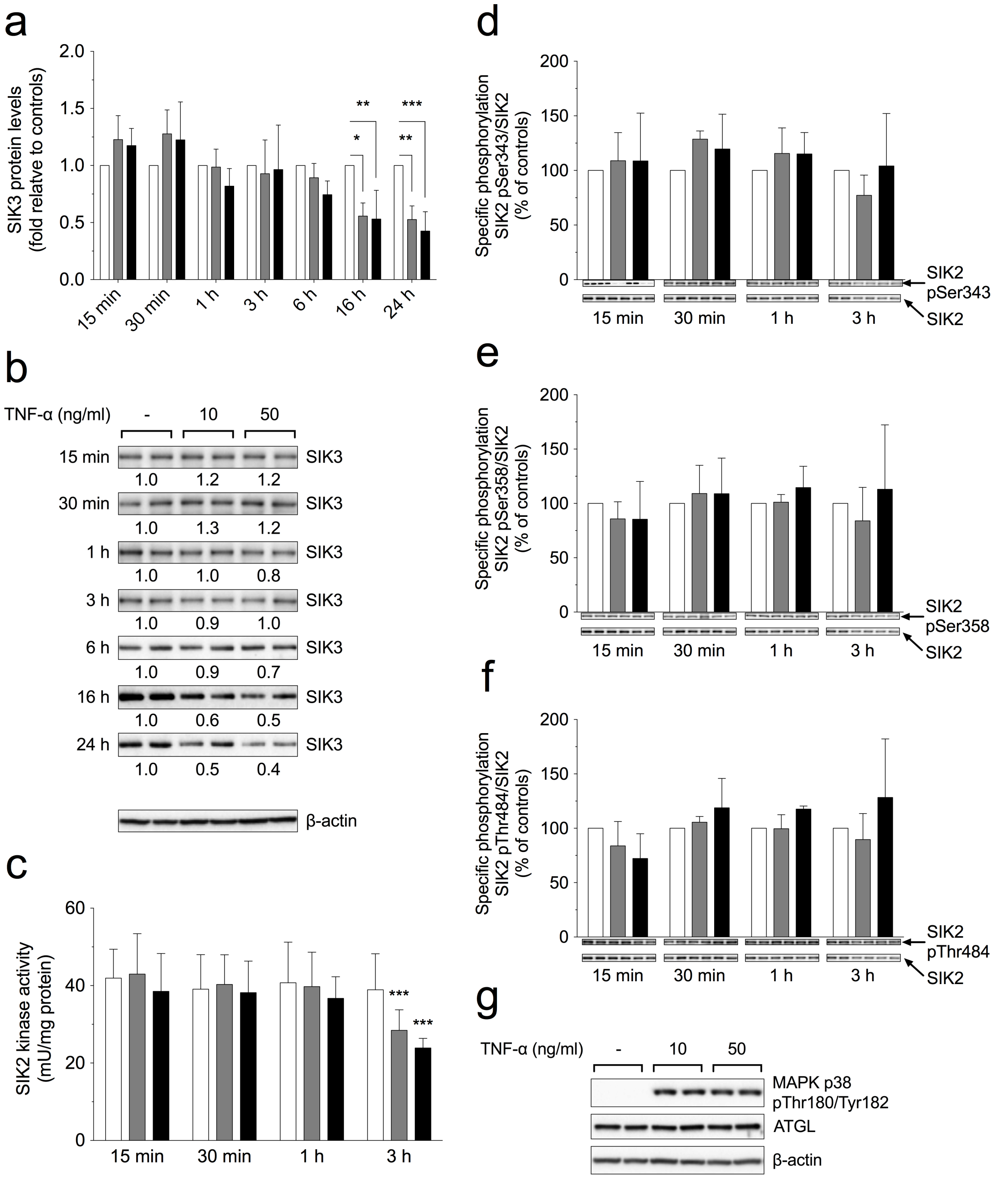

ESM Figure 3. ***Regulation of SIK3 protein levels, SIK2 kinase activity and SIK2 phosphorylation by TNF-α in adipocytes.*** 3T3-L1 adipocytes treated with 10 ng/ml (grey bars) or 50 ng/ml (black bars) TNF-α, or vehicle (white bars) for indicated time points (*n*=3 independent experiments). (**a**) SIK3 protein levels. (**b**) Representative blots with mean values for relative protein levels below. β-actin and HSP90 were used as loading controls. (**c**) SIK2 *in vitro* kinase activity against the peptide substrate HDAC5tide. (**d–f**) Phosphorylation of SIK2 at Ser343 (**d**), Ser358 (**e**) and Thr484 (**f**),

normalised to total SIK2. (**g**) Phosphorylation of MAPK p38 (Thr180/Tyr182) and protein levels of ATGL at 15 min and 24 hours of treatment, respectively. Statistical significance determined by two-way ANOVA with Tukey's multiple comparisons post hoc test (**a, c**). *$p<0.05$, **$p<0.01$, ***$p<0.001$

ESM Figure 4

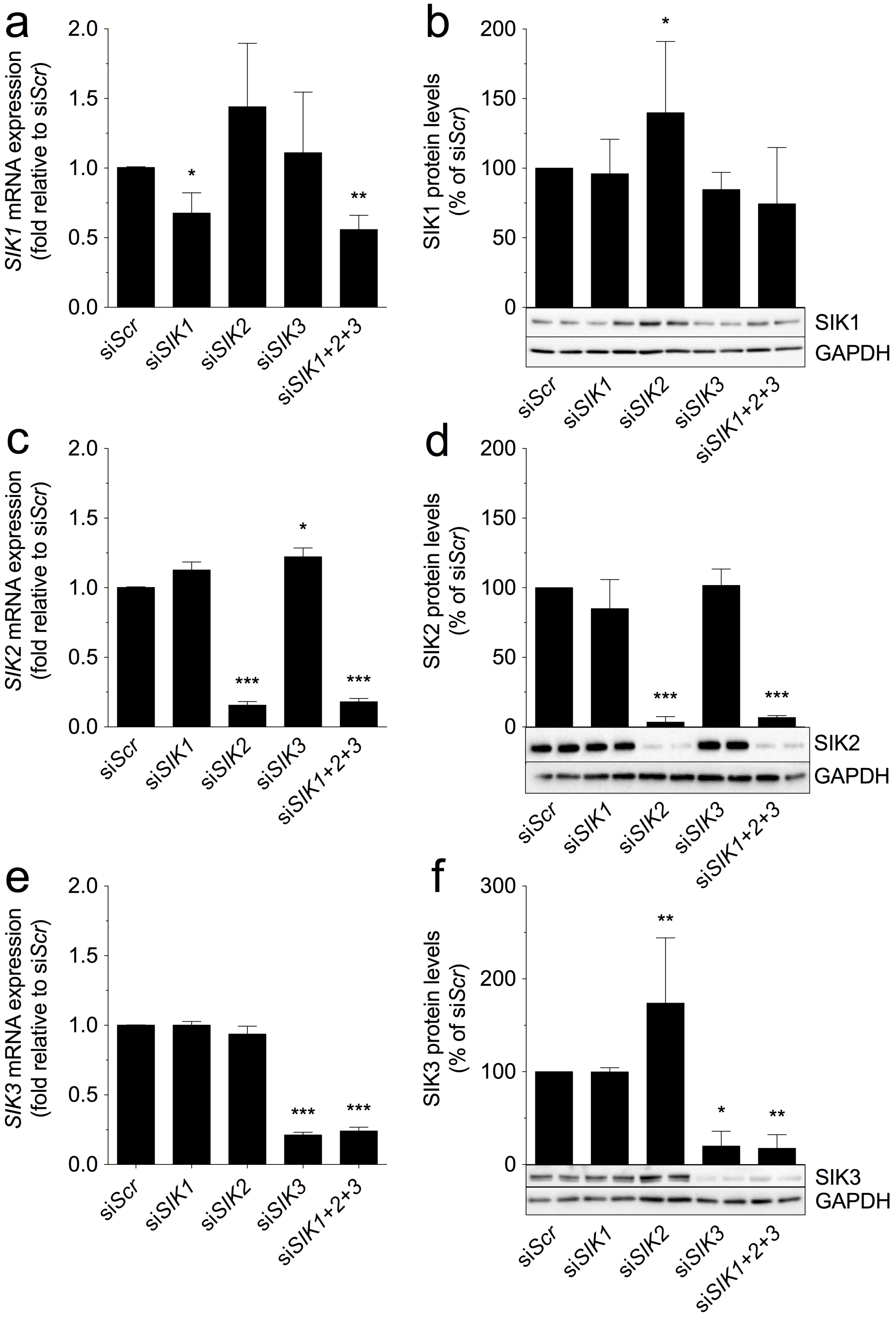

ESM Figure 4. ***Expression of SIKs after siRNA silencing.*** SIK isoforms were silenced using siRNA in *in vitro* differentiated human adipocytes (si*SIK1* *n*=4, si*SIK2*

n=4-8, si*SIK3* *n*=4 and si*SIK1+2+3* *n*=4 independent experiments). mRNA expression of *SIK1* (**a**), *SIK2* (**c**) and *SIK3* (**e**). *18S rRNA* was used as reference gene. Protein levels of SIK1 (**b**), SIK2 (**d**) and SIK3 (**f**). GAPDH was used as loading control. Statistical significance determined by one-way ANOVA with Dunnett's multiple comparisons post hoc test. *$p<0.05$, **$p<0.01$, ***$p<0.001$

ESM Figure 5

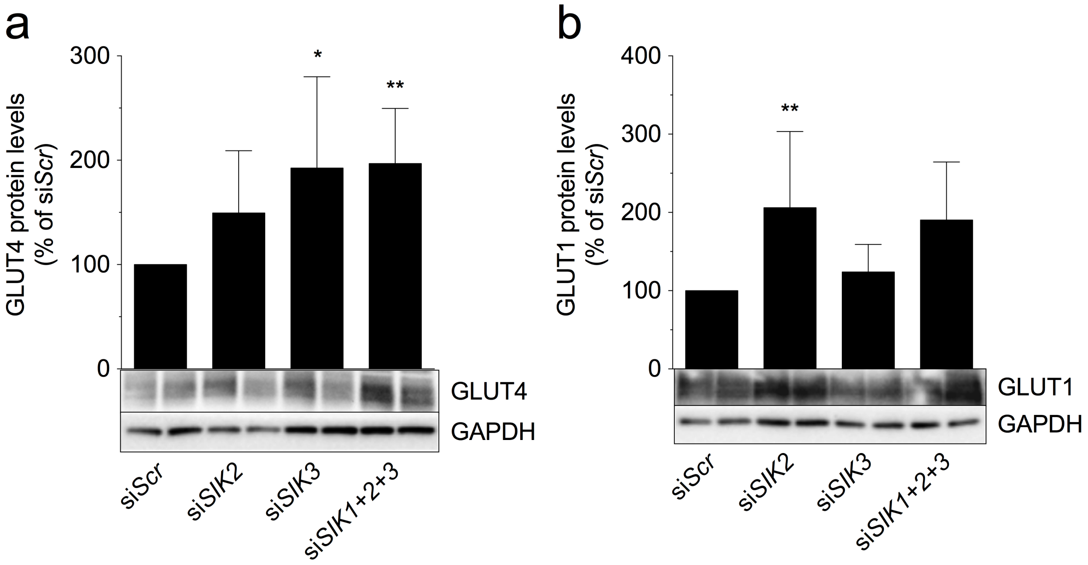

ESM Figure 5. ***GLUT1 and GLUT4 protein levels after silencing SIKs.***

SIK isoforms were silenced using siRNA in *in vitro* differentiated human adipocytes (si*SIK2* n=6-8, si*SIK3* n=4 and si*SIK1+2+3* n=4 independent experiments). Protein levels of GLUT4 (**a**) and GLUT1 (**b**). GAPDH was used as loading control. Statistical significance determined by one-way ANOVA with Dunnett's multiple comparisons post hoc test. $^*p<0.05$, $^{**}p<0.01$
